# Behçet’s syndrome-like features revealing myelodysplastic syndrome with *TP53* mutation: a case report

**DOI:** 10.3389/fimmu.2026.1803414

**Published:** 2026-04-01

**Authors:** Andrej Pesic, Jelena Ljubicic, Milena Todorovic Balint, Kristel Klaassen, Marina Andjelkovic, Sonja Pavlovic, Maja Stojanovic

**Affiliations:** 1 Clinic of Hematology, University Clinical Center of Serbia, Belgrade, Serbia; 2 Clinic of Allergy and Immunology, University Clinical Center of Serbia, Belgrade, Serbia; 3 Faculty of Medicine, University of Belgrade, Belgrade, Serbia; 4 Institute of Molecular Genetics and Genetical Engineering, University of Belgrade, Belgrade, Serbia

**Keywords:** Behçet’s syndrome, case report, myelodysplastic syndrome, SF3B1, TP53, whole-exome sequencing

## Abstract

The coexistence of Behçet’s syndrome (BS) and myelodysplastic neoplasm (MDS) is increasingly recognized and is often referred to as MDS with BS-like features. These patients demonstrate a distinctive profile characterized by a high prevalence of trisomy 8 and limited response to conventional immunosuppressive therapy. However, the mutational profile of this rare entity remains almost entirely unexplored. Here, we report a 36-year-old female presenting with painful oral and genital ulcers who is subsequently diagnosed with an atypical form of BS. Nonetheless, whole-exome sequencing identified monoallelic *TP53* and *SF3B1* mutations, findings highly indicative of MDS. After confirmation of diagnosis and prognostic assessment, the patient ultimately underwent successful allogeneic stem cell transplantation. This case highlights the importance of comprehensive genomic profiling in diagnosing, risk-stratifying, and managing patients with rare hematological entities presenting with autoinflammatory phenomena.

## 1 Introduction

Behçet’s syndrome (BS) is a complex, multifactorial inflammatory condition, characterized by heterogeneous clinical manifestations and a relapsing-remitting course. BS most commonly presents with recurrent oral and genital ulcers, skin lesions, ocular inflammation, and gastrointestinal involvement (1). On the other hand, myelodysplastic syndrome (MDS) - now termed myelodysplastic neoplasm, is a heterogeneous group of stem-cell disorders commonly manifesting as pancytopenia due to ineffective hematopoiesis (2).

The coexistence of BS and MDS has previously been documented (3, 4), and patients with MDS who develop BS-like symptoms are increasingly recognized as having MDS with BS-like features (BL-MDS) (5). Patients with BL-MDS frequently exhibit refractoriness to conventional immunosuppressive treatments and carry a poor prognosis (6). Haematopoietic stem cell transplantation (HSCT) is a promising treatment modality for these patients, as it represents the only potentially curative treatment for MDS, with a demonstrated efficacy in severe autoimmune and inflammatory disorders (7, 8). The genetic landscape plays a pivotal role in the management of patients with MDS, culminating in the development of the Molecular International Prognostic Scoring System (IPSS-M), upgrading the previous Revised International Prognostic Scoring System (IPSS-R) (9). Mutations in tumor protein p53 gene (*TP53*) have emerged as clinically most significant, despite occurring in only 5% to 10% of patients with *de novo* MDS and AML (10). In MDS, the presence of a *TP53* mutation is associated with high-risk disease, resistance to therapy, and poor outcomes, although the allelic state of the gene is critical for diagnostic and prognostic stratification (11). Despite the growing recognition of the central role of molecular features in MDS, the mutational profile of patients with BL-MDS remains almost entirely unexplored.

To our knowledge, *TP53* mutations have not been previously reported in this context. Here, we present the first documented case of a patient presenting with BS-like MDS harboring a *TP53* mutation, who was successfully treated with allogeneic stem cell transplantation. This case highlights the importance of comprehensive genomic profiling in diagnosing, risk-stratifying, and managing patients with MDS and atypical inflammatory manifestations.

## 2 Case presentation

A 36-year-old woman presented in February 2024. with fever and painful oral and genital ulcerations. During the previous year, the patient experienced recurrent episodes of fever of unknown origin, with a similar episode of spontaneously resolved painful genital ulcerations occurring two months earlier. Her medical history was notable for recurrent episodes of hidradenitis suppurativa, which required surgical intervention in 2022. Family history was significant for pancreatic cancer in her mother. She was initially evaluated by an infectious disease specialist, who excluded sexually transmitted infections and was subsequently referred to a clinical immunologist. Laboratory testing revealed markedly elevated inflammatory markers (CRP 118mg/L, (normal value (NV) < 5mg/L); ferritin 609 µg/L, (NV 4.6-204 µg/L)) and macrocytic anemia (Hgb 11g/dL (NV 12-16g/dL), MCV 114fL (NV 80-100fL)).

Given the patient’s presentation, a pathergy test was performed and was weakly positive, contributing to a total score of 5 points according to the latest International Criteria for Behçet’s Disease (ICBD) (12). Therefore, a comprehensive evaluation was initiated; however, HLA typing (HLA-B07/08) did not support a diagnosis of BS. Transthoracic echocardiography and ophthalmologic examination were unremarkable. Upper gastrointestinal endoscopy revealed mild inflammatory changes, which were confirmed by histopathology, while magnetic resonance enterography demonstrated inflammation of the gastric antrum, pylorus, and duodenum. An incidental but notable finding was a markedly elevated CA 72–4 level of 164.9 kU/L (NV<6.9 kU/L). After excluding other causes, this was attributed to gastrointestinal inflammation associated with BS.

The patient was initially treated with prednisone (0.5mg/kg/day), which led to marked clinical improvement, including resolution of fever, healing of ulcerative lesions, and normalization of inflammatory markers. Steroids were gradually tapered, and colchicine was added as an immunomodulatory agent; however, it was discontinued due to gastrointestinal intolerance, which also occurred with azathioprine, leaving the patient on prednisone monotherapy. Throughout the treatment, macrocytic anemia persisted (hemoglobin 10.5–11g/dL; MCV 114–117fL) as the only abnormality in the blood count. Common causes of macrocytosis, including folate and B12 deficiency, hemolysis, medications, hypothyroidism, liver disease, and alcohol abuse, were excluded.

**Figure 1:**
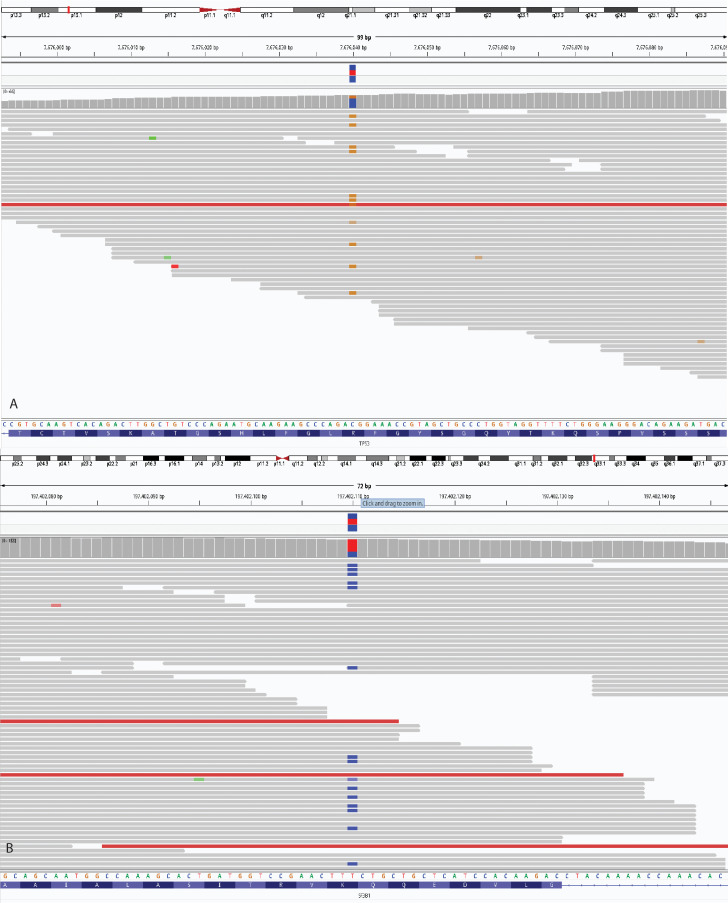
Integrative Genomics Viewer showing c.329G>C (p.Arg110Pro) variant in the *TP53* gene **(A)** and c.2098A>G p.(Lys700Glu) variant in the *SF3B1* gene **(B)**.

**Figure 2:**
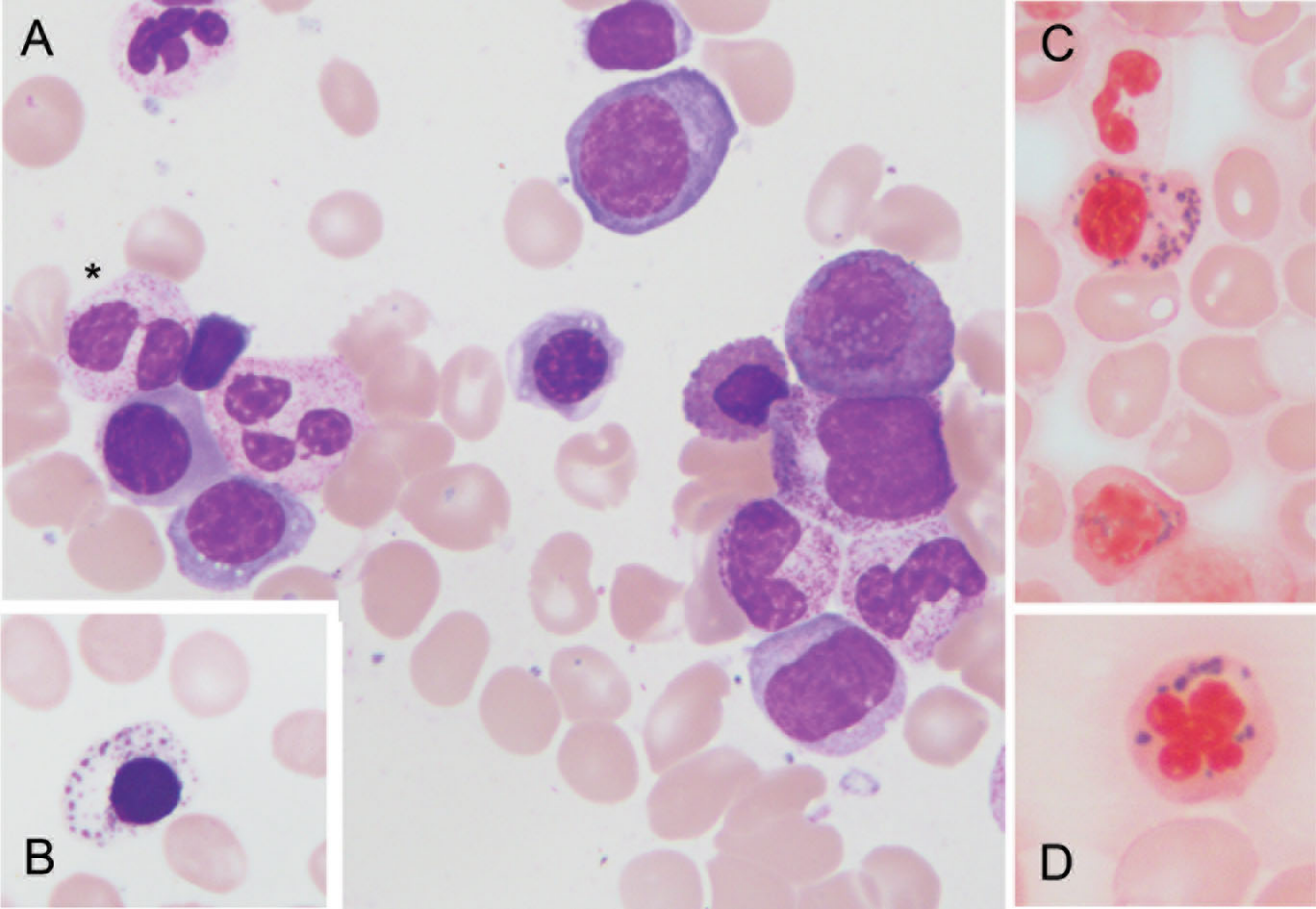
Overall appearance of dysplastic changes observed in our patient. Megaloblastic erythroblasts, some of them with small vacuolation. Star denotes pseudo “Pelger Huet” abnormality (May Grunwald Giemsa stain, 1000x) **(A)**; “Ghost like” erythroblast with basophilic iron deposits (May Grunwald Giemsa stain, 1000x) **(B)**; Atypical “ring” sideroblasts (Pearls stain, 1000x and 1200x) **(C, D)**.

Given the patient’s atypical clinical presentation and young age, genetic testing was pursued under the suspicion of a monogenic autoinflammatory disorder. Clinical exome sequencing did not reveal pathogenic variants associated with monogenic autoinflammatory syndromes or BS. However, it identified a pathogenic *TP53* variant, c.329G>C (p.Arg110Pro) (NM_000546.6), reported as an incidental finding. Subsequent whole-exome sequencing (WES) confirmed the *TP53* variant, with a variant allele frequency (VAF) of 0.26, and an additional pathogenic variant, c.2098A>G (p.Lys700Glu), in the *SF3B1* gene (NM_012433.4), with a VAF of 0.28 (**Figure 1**). The identification of a pathogenic *SF3B1* variant, commonly associated with MDS, in addition to the patient’s persistent macrocytic anemia, prompted referral for further hematologic evaluation. Bone marrow aspiration and biopsy were performed, revealing dysplastic cells in multiple lineages (**Figure 2**), while conventional cytogenetic analysis detected the presence of trisomy 8 in 80% of metaphases. Integrated with molecular findings, these results were consistent with a diagnosis of MDS with mutated *SF3B1* (MDS*-SF3B1*), according to International Consensus Classification (13).

The patient was initially stratified as low risk by both IPSS-R and IPSS-M. However, given the atypical presentation of MDS in a young patient, in addition to her mutational profile, allogeneic HSCT was considered as a treatment option. Accordingly, HLA typing of the family was performed, identifying the patient’s 38-year-old brother as a potential matched sibling donor (MSD). Given the patient’s positive family history for pancreatic cancer, in addition to the diagnosis of MDS at a young age (<40 years), a germline *TP53* mutation was excluded by performing WES from the patient’s buccal swab, as were other germline predisposition alleles. Furthermore, WES analysis of the brother’s peripheral blood, in addition to the patient’s mother and father, revealed no pathogenic predisposition alleles, including *TP53* and *SF3B1*. To manage the symptoms, the patient was maintained on low-dose prednisone with close clinical monitoring. HSCT was scheduled after the completion of oocyte cryopreservation.

However, only two months after making the diagnosis, the patient’s anemia significantly worsened (hemoglobin 7g/dL), and she became transfusion dependent, requiring two erythrocyte transfusions per month. A repeated bone marrow aspiration excluded elevated blast count. The patient’s IPSS-R was subsequently upgraded to intermediate, reinforcing the initial decision to proceed with transplantation.

Allogeneic HSCT from MSD was performed in May 2025. Upon admission, laboratory tests showed a WBC of 3x10^9/L, hemoglobin of 9.1 g/dL, and platelet count of 140x10^9/L. However, cytogenetic analysis repeated before transplantation demonstrated further clonal evolution to a complex karyotype, including deletions of chromosomes 2 and 5q in 8 of the 20 metaphases. Despite a normal blast count in the bone marrow aspirate, these findings reclassified the patient as high risk according to both the IPSS-R and IPSS-M. The conditioning regimen prior to HSCT included treosulfan, fludarabine, and melphalan, in combination with *in vivo* T-cell depletion using anti-T-lymphocyte globulin (ATLG; Grafalon^®^ 10 mg/kg from day -3 to day -1). Graft-versus-host disease (GvHD) prophylaxis included cyclosporine A (maintained at trough serum concentrations 100–150 ng/mL) and methotrexate (15 mg/m² on day +1 and 10 mg/m² on days +3 and +6). To promote myeloid engraftment, granulocyte colony-stimulating factor (G-CSF) was administered from day +5, with successful neutrophil engraftment documented on day +10. On day +16, the patient developed grade II acute cutaneous GvHD, which was successfully treated with corticosteroids.

**Figure 3:**
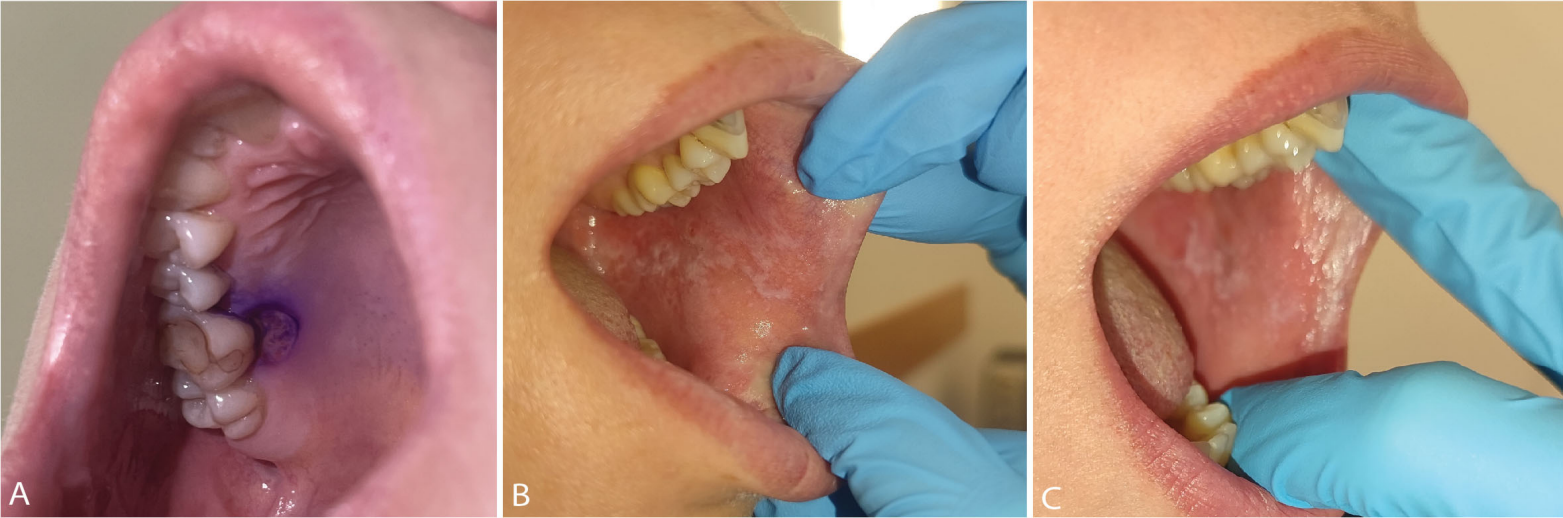
Atypical oral ulceration at initial presentation stained with Gentian-violet **(A)**; Chronic graft-versus-host disease localized in the oral mucosa, before **(B)** and after **(C)** treatment with local immunosuppressive therapy.

**Figure 4:**
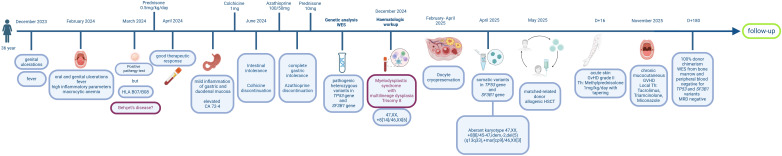
Clinical course of the patient. HLA, Human Leukocyte Antigen; WES, Whole Exome Sequencing; HSCT, Haematopoietic stem cell transplantation; GvHD, graft-versus-host disease; MRD, Measurable Residual Disease. Created in BioRender. Ljubicic, J. (2026) https://BioRender.com/n9fg64p and licensed under CC BY 4.0.

On day +28, hematologic reevaluation revealed complete hematologic remission, a normal male karyotype, and full donor chimerism. Cyclosporine A was gradually tapered and discontinued four months post-transplantation. Next-generation sequencing (NGS) testing of bone marrow and peripheral blood at days +90 and +180 confirmed clearance of pathogenic *TP53* and *SF3B1* variants (**Supplementary Figure 1**). Eight months after HSCT, the patient remains in complete remission, with no BS-like features and a mild form of chronic GvHD limited to the oral mucosa treated with local immunosuppressive treatment, shown in the **Figure 3**. The most important clinical events of the patient’s timeline are shown in **Figure 4**.

## 3 Genetic analysis

Genomic DNA was isolated from whole blood using QIAamp DNA-Blood-Mini-Kit (QIAGEN, Germany) and initially analyzed by clinical exome sequencing (CES) with TruSight one gene panel, comprising 4813 known disease-associated genes (Illumina, San Diego, CA, USA). Library preparation was done using 50 ng of genomic DNA according to Illumina DNA Prep with Enrichment protocol. Sequencing (with 150-bp paired-end runs) was performed on Illumina NextSeq 550 System (Illumina, San Diego, CA, USA), with alignment and analysis performed against the GRCh38/hg38 reference genome assembly. Variants passing quality call (QC) filters (quality score >Q20, read depth >20, percentage of variant frequency for the minor allele >20%) and with minor allele frequencies <1% in The Genome Aggregation Database (gnomAD) were further analyzed. Systematic interpretation of variants was performed using VarSome Clinical (Saphetor, Lausanne, Switzerland). All variants detected were classified using American College of Medical Genetics and Genomics (ACMG) guidelines (Richards et al., 2015). WES was performed using 50 ng of genomic DNA according to Illumina DNA Prep with Exome 2.0 Plus Enrichment kit protocol, while sequencing was performed on Illumina NextSeq 2000 System (Illumina, San Diego, CA, USA), with alignment and analysis performed as described for CES.

## 4 Discussion

In this article, we report the case of a 36-year-old female patient who presented with features resembling BS in the context of underlying MDS. NGS revealed pathogenic variants strongly associated with MDS, triggering further workup that confirmed the diagnosis, with an additional important role in the therapeutic management. Patients with BL-MDS show distinct clinical features, such as a markedly higher prevalence of trisomy 8 (76.5% compared to 7–9% in patients with MDS alone) (4), a female predominance, more frequent gastrointestinal involvement, and fewer ocular manifestations compared to classic BS (14). These characteristics are consistent with the clinical presentation observed in our case. Manifestations of BL-MDS are believed to be mediated by abnormal neutrophils and monocytes harboring trisomy 8, as several genes associated with immunity and inflammation are overexpressed in patients having this aberration (15, 16). The age of initial symptom onset varies significantly, ranging from 4 to 82 years (3, 17). In the majority of reported cases, manifestations resembling BS, most commonly oral and gastrointestinal lesions, precede the detection of MDS by approximately 2 to 10 years (18). A simultaneous diagnosis occurs in one-third of patients, while a diagnosis of MDS prior to the appearance of BS-like features is the least frequent scenario (8). In our case, there was a documented history of a mild macrocytic anemia occurring five years prior to the onset of genital ulcerations. At that time, diagnostic evaluation was limited only to assessing vitamin B12 and folate levels. Retrospectively, this finding likely represented the first manifestation of her disease, emphasising that macrocytosis is often the earliest recognizable feature of MDS. It can particularly be overlooked in patients with BS-like disease, as it can be associated with azathioprine use, which is common in this setting, or with malabsorption due to gastrointestinal involvement by the disease (6).

Previous studies have concluded that HSCT remains the only curative treatment option for high-risk MDS patients presenting with intestinal form of BL-MDS (8). However, molecular characterization of these patients is lacking. Given the relatively recent integration of NGS sequencing into the diagnostic workup for MDS, it is possible that some of the previously reported cases shared a similar mutational profile to ours, but were not molecularly characterized. Indeed, NGS analysis was described in only three cases of BL-MDS, revealing heterozygous variants in *PTPN11*, *MEFV* and *GATA2* genes (3, 17, 19). Among these, only two patients ultimately underwent successful HSCT, with neither case guided directly by the molecular findings. In contrast, the identification of pathogenic variants in *TP53* and *SF3B1* played a pivotal role in indicating the diagnosis, guiding subsequent risk stratification, and enabling a personalized decision to proceed with transplantation in our patient (18, 20–22). Only the recent study by Ding et al. has systematically addressed the genetic background of BS-like features associated with trisomy 8, with neither case harboring a *TP53* mutation (23). Finally, there remains a possibility that cases of MDS with *TP53* mutations presenting with similar autoinflammatory phenomena may exist in the literature, although not specifically labeled as BL-MDS.

In order to determine the treatment approach and prognosis, it was essential to characterize our patient’s *TP53* status. The *TP53* allelic state (single or multi-hit), along with VAF, the position of mutation within the clonal hierarchy, mutation type, and the presence of co-occurring mutations, collectively define genetic heterogeneity within MDS patients. This influences outcomes and tailors the selection of patients who are candidates for HSCT (24). According to the International Consensus Classification, criteria for a multi-hit (biallelic) *TP53* mutation include the presence of two or more mutations, each with a VAF of 10% or greater, or a single mutation with a VAF greater than 50%, and/or a VAF greater than or equal to 10% with evidence of copy-neutral loss of heterozygosity (CN-LOH) (25). As previously described, NGS identified a single *TP53* mutation with a VAF of 26% and no evidence of CN-LOH, while karyotype analysis failed to detect 17p deletion. Consequently, our patient was classified as having a single-hit (monoallelic) *TP53* mutation. Accordingly, the patient was diagnosed as having MDS with mutated *SF3B1*, as multi-hit *TP53* status is required for the diagnosis of *TP53*-mutated MDS (13). The distinction between these forms of MDS is crucial, as the latter form is associated with a more aggressive, rapidly progressive disease (26). These patients typically have poor outcomes even after allogeneic HSCT, with no therapies showing promising results to date (24, 27, 28). Patients with multi-hit *TP53* have lower overall survival and higher rates of transformation to AML when compared to single-hit patients, regardless of the IPSS-R risk (11, 29, 30). Although single-hit mutations generally correlate with outcomes similar to those of wild-type TP53, prognosis remains heterogeneous. Outcomes vary based on the presence of co-occurring mutations, with patients lacking other mutations demonstrating a more favorable prognosis (11, 24). However, the majority of monoallelic patients, including our case, carry at least one additional mutation, such as *SF3B1*. In addition to the allelic state, *TP53* VAF has also been demonstrated to have prognostic significance in MDS (10, 24, 31–33). The strong correlation between high VAF and multi-hit disease partially explains this, as a VAF greater than 50% essentially defines a multi-hit status. Accordingly, most studies have identified VAF values near 50% as predictors of worse outcomes. However, the only study specifically evaluating the impact of VAF in single-hit patients found that it can predict high-risk disease at much lower levels. In this specific study, patients with VAF >22% had worse overall survival, while those with VAF <22% had outcomes comparable to wild-type patients (11). In this context, our patient’s VAF of 26% could have implicated a more aggressive disease, although this conclusion comes from a subgroup analysis, and more studies are needed to solidify the VAF threshold that confers higher-risk disease in patients with single-hit mutations. Furthermore, other studies have shown that the benefit of HSCT is most evident in patients having *TP53* VAF less than 40%, 45% and 50%, respectively, although patients with both MDS and AML were included (33–35). Finally, certain *TP53* mutation hotspots, such as the ones in codons 175 and 248, have been associated with poor prognosis in monoallelic patients, although none of these variants were identified in our case (11, 36).

Data from the literature indicate better outcomes for HSCT in patients with single-hit compared to those with multi-hit mutations (11, 32). In addition to these findings, other high-risk features of our patient, such as the presence of BS-like features, R-IPSS score, an *SF3B1* co-mutation, and a moderately high *TP53* VAF, the decision to proceed to HSCT seemed appropriate. Unfortunately, despite the availability of a related donor, the process of cryopreservation significantly delayed the timing of HSCT. Even though the patient’s blood count remained stable at the time of transplantation and the bone marrow aspirate showed no increase in blasts, cytogenetic analysis revealed clonal evolution to a complex karyotype. This finding significantly altered the prognosis of our patient, as patients with a complex karyotype are classified as a very high-risk group, although the outcomes are still better compared to patients with multi-hit *TP53* mutation (31, 37). Despite the evidence of clonal evolution, we chose not to increase the intensity of the conditioning regimen, as *TP53* mutations are known to confer chemoresistance, which is in line with published data failing to demonstrate the benefit of myeloablative conditioning compared to reduced-intensity conditioning in patients with MDS and mutated *TP53* (27, 29, 32). We also decided to proceed with a matched related donor, since the use of this donor type has previously been shown to improve survival in these patients (31). Given that early disease relapse is the leading cause of treatment failure following HSCT, we decided to initiate a very early tapering of immunosuppression. This decision was based on evidence that the development of chronic GvHD associates with a reduced risk of post-HCT relapse in these patients, indicating susceptibility to a graft-versus-leukemia (GvL) effect (32, 38). Therefore, cyclosporine A was completely discontinued four months after HSCT, despite the patient having previously experienced acute GvHD. This decision likely played a significant role in the development of chronic GvHD, which, although mild, precluded us from administering prophylactic donor lymphocyte infusions. Interestingly, chronic GvHD was localized exclusively in the oral mucosa, which was previously affected by BS-like manifestations (**Figure 3**). Since manifestations of BL-MDS are thought to be mediated by inflammatory cells derived from the abnormal clone, the localized nature of GvHD may reflect increased immunological activity at a site where GvL was amplified. We believe this significantly contributed to our patient remaining in a measurable residual disease-negative remission to date.

The main limitation of our study is the fact that NGS was not repeated prior to transplantation. This was primarily due to limited access to the necessary analysis and the fact that the patient had not received any specific treatment since the previous testing. Moreover, monoallelic *TP53* mutations usually represent late subclonal events within clones already carrying co-mutations, whereas biallelic mutations generally occur earlier and give rise to dominant clones with less frequent co-mutations (11, 39). These factors, in addition to a relatively short interval of only a few months since the previous analysis and the absence of chemotherapy, which could have exerted a selective pressure on the *TP53* mutated clone, argue against the development of a multi-hit disease during this period. However, other elements could favor the emergence of a multi-hit clone, such as worsening of anemia, and, most importantly, clonal evolution to a complex karyotype. This ultimately precludes a definitive conclusion on whether our patient developed multi-hit disease prior to transplantation.

## 5 Conclusion

In conclusion, we report the case of a patient with myelodysplastic neoplasm who presented with features resembling Behçet’s syndrome and was successfully treated with allogeneic stem cell transplantation, primarily due to the availability of comprehensive genomic profiling. Whole-exome sequencing was instrumental in revealing mutations strongly indicative of MDS, prompting confirmatory workup and subsequent therapeutic strategy. This case highlights the critical role of advanced molecular diagnostics in enabling precision medicine for conditions that would otherwise remain unrecognized or undertreated, favorably altering the patient’s clinical outcome.

## Data Availability

The original contributions presented in the study are included in the article/**Supplementary Material**. Further inquiries can be directed to the corresponding author.

## Supplementary material

Supplementary Figure 1 Integrative Genomics Viewer from bone marrow showing the regions of the *TP53* gene **(A)** and *SF3B1* gene **(B)** with wild-type sequence six months after transplantation.
