# Supplementary figures and images for: Behçet’s syndrome-like features revealing myelodysplastic syndrome with TP53 mutation: a case report
Source: Front Immunol. 2026 Apr 1;17:1803414. doi: 10.3389/fimmu.2026.1803414 (PMC13079325; doi:10.3389/fimmu.2026.1803414)

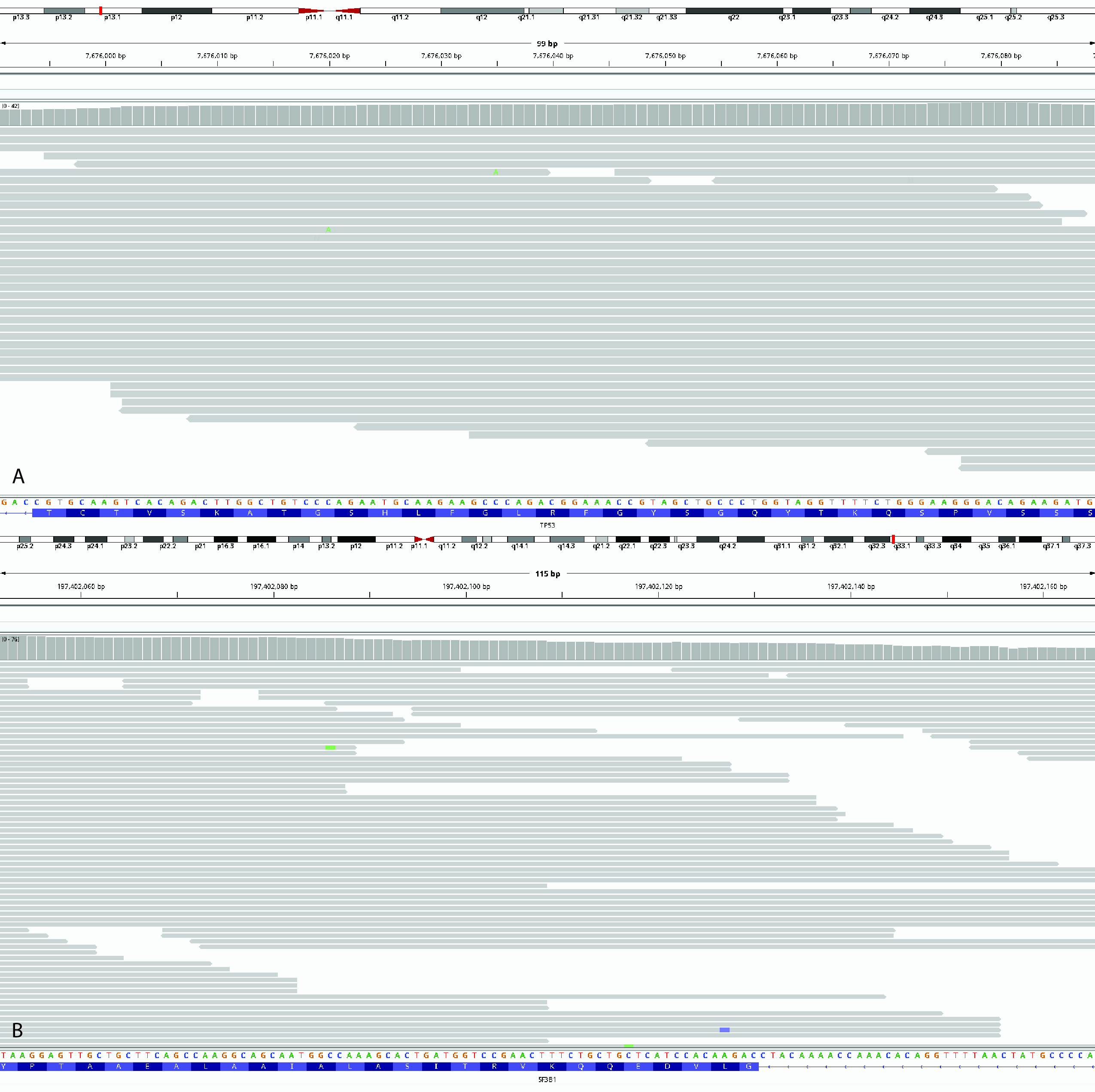

Supplement: Supplementary Figure 1 — Integrative Genomics Viewer from bone marrow showing the regions of the TP53 gene (A) and SF3B1 gene (B) with wild-type sequence six months after transplantation. [file Image1.jpeg]
